# PSORS1 Locus Genotyping Profile in Psoriasis: A Pilot Case-Control Study

**DOI:** 10.3390/diagnostics12051035

**Published:** 2022-04-20

**Authors:** Noha Z. Tawfik, Hoda Y. Abdallah, Ranya Hassan, Alaa Hosny, Dina E. Ghanem, Aya Adel, Mona A. Atwa

**Affiliations:** 1 Dermatology, Venereology and Andrology Department, Faculty of Medicine, Suez Canal University, Ismailia 41522, Egypt; atwamona@gmail.com; 2 Medical Genetics Unit, Histology & Cell Biology Department, Faculty of Medicine, Suez Canal University, Ismailia 41522, Egypt; hoda_ibrahim1@med.suez.edu.eg; 3 Center of Excellence in Molecular and Cellular Medicine, Faculty of Medicine, Suez Canal University, Ismailia 41522, Egypt; 4 Clinical Pathology Department, Faculty of Medicine, Suez Canal University, Ismailia 41522, Egypt; rania.moustafa@med.suez.edu.eg; 5 Ministry of Health, Cairo 11435, Egypt; eng_aliuop@yahoo.com (A.H.); drdinaghanem@gmail.com (D.E.G.); ayadel90@gmail.com (A.A.)

**Keywords:** psoriasis, *PSORS1C3*, *PSORS1C1/CDSN*, *LOC105375015*, rs1062470, rs887466, rs10484554, single-nucleotide polymorphism

## Abstract

(1) Background: The psoriasis susceptibility 1 (PSORS1) locus, located within the major histocompatibility complex, is one of the main genetic determinants for psoriasis, the genotyping profile for three single-nucleotide polymorphisms (SNPs) comprising the PSORS1 locus: rs1062470 within *PSORS1C1/CDSN* genes, rs887466 within *PSORS1C3* gene, rs10484554 within *LOC105375015* gene, were investigated and correlated with psoriasis risk and severity. (2) Methods: This pilot case-controlled study involved 100 psoriatic patients and 100 healthy individuals. We investigated three SNPs and assessed the relative gene expression profile for the *PSORS1C1* gene. We then correlated the results with both disease risk and severity. (3) Results: The most significantly associated SNP in PSORS1 locus with psoriasis was rs10484554 with its C/T genotype 5.63 times more likely to develop psoriasis under codominant comparison. Furthermore, C/T and T/T genotypes were 5 times more likely to develop psoriasis. The T allele was 3 times more likely to develop psoriasis under allelic comparison. The relative gene expression of *PSORS1C1* for psoriatic patients showed to be under-expressed compared to normal controls. (4) Conclusions: Our study revealed the association of the three studied SNPs with psoriasis risk and severity in an Egyptian cohort, indicating that rs10484554 could be the major key player in the PSORS1 locus.

## 1. Introduction

Psoriasis is a common inflammatory skin disease of multifactorial origin that causes significant stress and morbidity [1]. It most often presents with well-demarcated, scaling and erythematous plaques, often at the extensor surfaces of knees and elbows [2]. Until now, the definite etiopathogenesis of psoriasis is not fully understood, however, it is widely regarded as a multifactorial disorder caused by the interaction between inherited susceptibility alleles and environmental triggers (e.g., stress, mechanical trauma and streptococcal infections) in combination with skin barrier disruption and immune dysfunction [3,4]. Recent advancements for expanding our understanding of psoriasis pathophysiology and targeted therapies are currently a hot topic in research [5,6].

Familial recurrence is also well documented and disease concordance is higher in monozygotic vs. dizygotic twins [7]. The main genetic determinant for psoriasis is the psoriasis susceptibility 1 locus (PSORS1), located within the major histocompatibility complex (MHC) on chromosome 6p21.3 [8] spanning from 180 to 250 kb [9,10]. The PSORS1 locus contains several genes, including protein-coding genes, non-protein-coding genes and pseudogenes. It has been found that some variants of them are associated with psoriasis [10,11,12,13].

**Figure 1:**
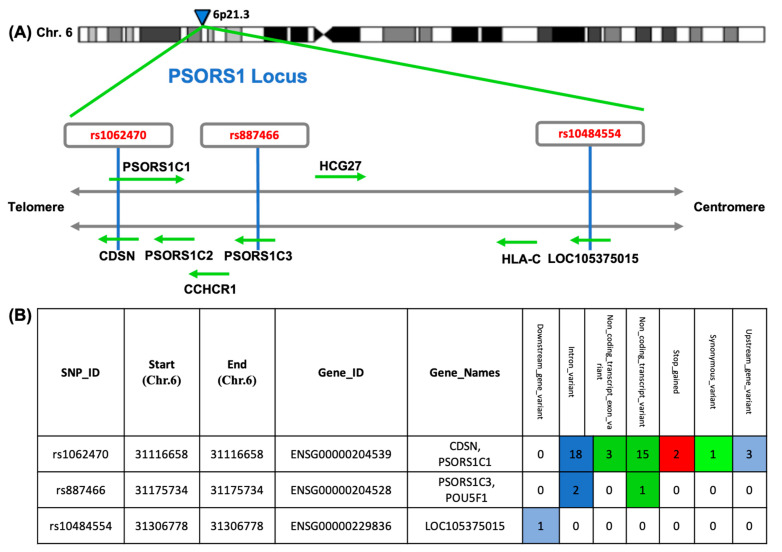
Single nucleotide polymorphisms (SNPs) included in the study. (**A**) Localization of the PSORS1 locus on human chromosome 6. (**B**) List of SNPs in the study to their gene names (official and Ensembl) with chromosomal coordinates and predicted variant effects. The variant effects are described with a color-coded set of variant consequences terms, defined by the sequence ontology and ordered by severity. The SNPs under study have 7 categories which are: downstream gene variant, intron variant, non-coding transcript exon variant, non-coding transcript variant, stop gained, synonymous variant, and upstream gene variant. (This diagram was constructed based on Ensembl https://www.ensembl.org/index.html (accessed on 1 March 2022) [15] and g:profiler tools https://biit.cs.ut.ee/gprofiler/gost (accessed on 1 March 2022) [16].

Single-nucleotide polymorphisms (SNPs) are substitutions of a single nucleotide at a specific position in the genome, which is present in at least 1% of the population [14] and may act as biomarkers for various complex diseases [15]. There are over 500 SNPs related to the PSORS1 locus [16]. Among all those variants, the following three SNPs have been suggested to be associated with psoriasis, namely: rs1062470, rs887466, and rs10484554. These SNPs are located at different points of the PSORS1 locus, as shown in Figure 1.

To date, and to the best of our knowledge, no data has been reported about PSORS1 locus SNPs among any Egyptian cohort, specifically for rs1062470, rs887466 and rs10484554 genetic variants as possible risk factors for psoriasis. Therefore, this study is the first to provide data about the association between these SNPs and psoriasis predisposition in the Egyptian population. This might help in anticipating the disease and early prophylactic measures could be taken.

## 2. Materials and Methods

A case-control study was conducted on two hundred participants. Written informed consent was taken from each patient before enrollment in the study. The study participants were divided into two groups: 100 Egyptian patients diagnosed with chronic plaque psoriasis of both genders with ages above 16 years old were recruited from the Dermatology Outpatient Clinics, and we excluded patients with psoriatic arthritis (PsA) or autoimmune diseases; and 100 healthy non-related participants of Egyptian descent, matched by age and gender to the patients with no family history of psoriasis or autoimmune diseases. All patients were subjected to full history taking and detailed dermatological examination. The severity of psoriasis was assessed using a PASI score that included an assessment of four body areas: head and neck (H), upper limbs (UL), trunk (T) and lower limbs (LL). Within each area, the severity of three signs, erythema (E), thickness/induration (I) and desquamation/scaling (D), is each assessed on a five-point scale: 0, none; 1, mild; 2, moderate; 3, severe; 4, very severe. According to the European consensus, interpretation of PASI is mild if the PASI score is <10, moderate if the PASI score is 10–20 and severe if PASI is >20 [17]. This study was performed in compliance with the guidelines of the Helsinki Declaration, 2013. Approval was taken from the Research Ethics Committee and the Institutional Review Board.

### 2.1. SNP Selection

The three studies’ SNPs were selected based on the level of evidence demonstrated by the number of publications studied, with each SNP adopted from https://opensnp.org/ (accessed on 1 March 2022) [18]. The rs10484554 exhibited a high level of evidence equivalent to 37 publications, while rs1062470 and rs887466 values were 4 and 7 publications, respectively. Our selection was also based on the latest findings of Wiśniewski et al. [19], who studied the same SNPs with a proven significance in psoriatic Poland patients, supported by the fact that these SNPs were not investigated among Egyptians in any published research.

### 2.2. Molecular Analysis

Lab work was performed in the Center of Excellence in Molecular and Cellular Medicine & Genetics Unit using three milliliters of venous blood in an EDTA anticoagulant vacutainer. They were kept at −20 °C till DNA extraction was performed.

### 2.3. DNA Extraction

Genomic DNA was extracted using the Invitrogen Gene Catcher purification system (Thermofisher, Waltham, MA, USA) from the frozen venous blood according to the manufacturer’s instructions. DNA concentration and purity were determined using a NanoDrop 2000 1C spectrophotometer (NanoDrop Tech., Inc. Wilmington, DE, USA).

### 2.4. Allelic Discrimination Analysis

The chosen genetic variants, rs1062470 (C__2438414_20), rs887466 (C___8941351_1) and rs10484554 (C__29612773_30) were genotyped using the TaqMan SNP Genotyping Assays (Thermofisher, Foster City, CA, USA) according to manufacturer’s instructions. The Applied Biosystems StepOnePlus Real-Time PCR detection system was used to conduct reactions and allelic discrimination, respectively.

### 2.5. PSORS1C1 Relative Gene Expression Analysis

Total RNA was extracted from the plasma of psoriatic patients and controls using the Qiagen miRNeasy mini kit (Qiagen, Hilden, Germany, Cat. no. 217004) following the protocol supplied by the manufacturer. RNA purity and concentration were assessed by a NanoDrop 2000 1C spectrophotometer (NanoDrop Tech., Inc. Wilmington, DE, USA). Complementary DNA (cDNA) was generated from total RNA with the miScript II RT Kit (Qiagen, Cat. no. 218161) in which *PSORS1C1* was polyadenylated by poly (A) polymerase and converted into cDNA by reverse transcriptase with oligo-dT priming. RT was carried out in a Veriti™ 96-Well Thermal Cycler (Applied Biosystems, Bedford, MA, USA) at 37 °C for 1 h, followed by inactivation of the reaction by briefly incubating at 95 °C. *GAPDH* was used as the endogenous control where it exhibited a uniform and stable expression in plasma samples with no significant difference between psoriatic patients and controls. Triplicate PCR reactions were carried out in the StepOne Real-Time PCR system (Applied Biosystems) using the miScript SYBR Green PCR Kit (Qiagen, cat. no 218076) and specific *PSORS1C1* primers: forward primer 5′-CTGACCGACTTTGCCACATGGA-3′, reverse primer 5′-GTGGGAAGAGGGAACCAGGATA-3′ and *GAPDH* primers: forward primer: 5′-GGAGCGAGATCCCTCCAAAAT-3′, reverse primer 5′-GGCTGTTGTCATACTTCTCATGG-3′ with negative controls in each run to exclude amplicon contamination.

The expression levels were done according to the quantitative real-time PCR experiments with minimal information required for publication (MIQE) guidelines. The relative *PSORS1C1* expression levels were calculated using the LIVAK method 2^(−ΔΔCq)^ [20], where Delta–Delta quantitative cycle (C_q_) = (C_q_ *PSORS1C1* − C_q_ *GAPDH*) _Psoriasis_ − (C_q_ *PSORS1C1* − C_q_ *GAPDH*) _controls_. The PCR ran initially at 95 °C for 5 min, followed by 40 cycles at 95 °C (15 s), then at 55 °C (1 min), and finally at 72 °C (1 min) for denaturation, annealing and elongation, respectively.

### 2.6. Statistical Analysis

Data were analyzed using the Statistical Package for the Social Sciences (SPSS), version 20.0 software, and GraphPad Prism version 7.0. Quantitative data were expressed as means ± standard deviation, while qualitative data were expressed as numbers and percentages. Two-sided Chi-square, Student-t, and ANOVA tests were used for parametric data. A *p*-value of <0.05 was considered statistically significant. Analyses of allele frequencies (number of copies of a specific allele divided by the total number of alleles in the group) and carriage rates (number of individuals with at least one copy of the A allele divided by the total number of individuals within the group) was carried out. Genotype frequencies were assessed for deviation from the Hardy–Weinberg equation by the online program (https://www.snpstats.net) (accessed on 1 March 2022) [21]. The relationship between allele frequencies and the presence of psoriasis was determined under different genetic association models using odds ratio with multiple logistic regression analysis after adjustment for psoriasis risk factors was investigated using the same program.

## 3. Results

### 3.1. Baseline Characteristics of the Study Population

The age of the patients and controls ranged from 18.0 to 60 years and 20.0 to 62.0 years, respectively, with no statistically significant difference between both groups. Regarding special habits, 77% of patients and 72% of controls were non-smokers. Concerning body mass index (BMI), the mean BMI was 26.80 ± 3.99 kg/m^2^ for patients, while it was 27.70 ± 3.93 kg/m^2^ in controls Table 1.

### 3.2. Clinical Assessment of Psoriasis Patients

The mean age of disease onset was 35.07 ± 13.43 and the mean duration was 6.75 ± 6.22 years. According to the age of disease onset, the patients were divided into three subgroups: (I) very early-onset psoriasis (vEOP): up to 20 years (21 patients); (II) middle early-onset psoriasis (mEOP): between 21 and 40 years (42 patients); late-onset psoriasis (LOP): above 40 years (37 patients). Forty-five percent of patients showed mild severity, 31% showed moderate severity, and 24% were severe (Table 2). There was a statistically significant difference between the age of onset of psoriasis in subgroups and gender; the vEOP group showed a higher percentage of females, while the median EOP and late EOP groups showed a higher percentage of males. There was no statistically significant difference between the age of onset of psoriasis in groups and the PASI score (Table 3).

### 3.3. Allelic Discrimination Analysis

**Figure 2:**
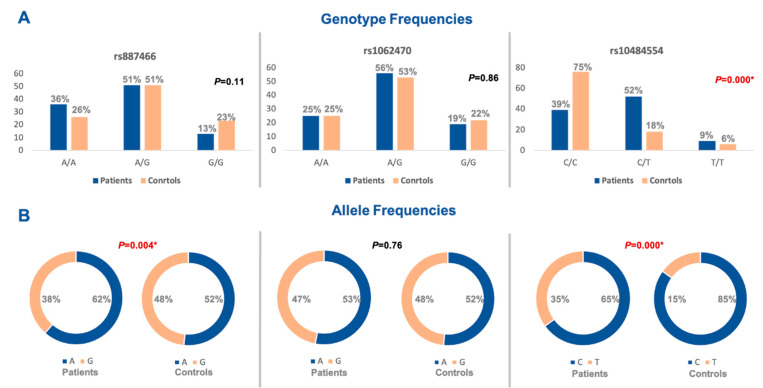
Genotype and allele frequencies of the studied genetic variants for the *PSORS1C3* gene. (**A**) Genotype frequencies of polymorphisms. (**B**) Allele frequencies of polymorphisms. A Chi-square test was applied. Statistical significance was set at *p* < 0.05. Bold red values with * indicate significant value.

The three studied polymorphisms were in accordance with Hardy–Weinberg equilibrium (rs887466: *p* = 0.53, rs1062470: *p* = 0.31, rs10484554: *p* = 0.19). On comparing the genotype frequency among the two study groups for rs887466 and rs1062470 genotypes, there was no statistically significant difference between patients and controls. On the contrary, the rs10484554 genotype showed a statistically significant difference between both groups (Figure 2A). Regarding variants comparison between the two study groups, the rs887466 A variant was more frequent among patients (62% in patients versus 52% in the control group), and also for the rs10484554 variant T, which was more frequent among patients (35% in patients versus 15% in the control group). Meanwhile, rs1062470 variants did not show a statistical difference between patients and controls (Figure 2B).

**Figure 3:**
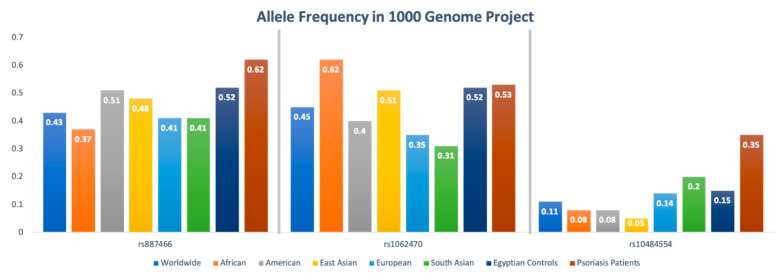
Allele frequencies of *PSORS1C3* gene rs887466, rs1062470 and rs10484554 in 1000Genome Project. This diagram was constructed based on Ensembl https://www.ensembl.org/index.html (accessed on 1 March 2022) [15].

For psoriatic patients, rs887466, rs1062470 and rs10484554 overall minor allele frequencies were 0.62 (A), 0.53 (A), and 0.35 (T), respectively. For controls, rs887466, rs1062470 and rs10484554 overall minor allele frequencies were 0.52 (A), 0.52 (A), and 0.15 (T), respectively. A comparison with other ethnic populations from the 1000Genome Project is shown in Figure 3.

### 3.4. Association of PSORS1 Locus Gene Variants with Psoriasis Risk

The rs887466 genotype G/G was 0.4 times more likely to protect against psoriasis under a codominant comparison (OR = 0.4, 95% CI = 0.17 to 0.95) and recessive model (OR = 0.5, 95% CI = 0.23 to 1.05). Moreover, allele G, for this polymorphism, was 0.66 times more likely to protect against psoriasis under allelic comparison (OR = 0.66, 95% CI = 0.44 to 0.99).

For the rs1062470 genotype, only A/G was 1.84 times more likely to develop psoriasis under over-dominant comparison (OR = 1.84, 95% CI = 1.09 to 3.13); other genotypes did not show a significant effect on disease risk (Table 4).

The rs10484554 genotype C/T was 5.63 times more likely to develop psoriasis under codominant comparison (OR = 5.63, 95% CI = 2.9 to 10.9). Moreover, the TT genotype was 2.92 times more likely to develop psoriasis under codominant comparison (OR = 2.92, 95% CI = 0.97 to 8.8). Considering the dominant comparison model, C/T and T/T genotypes were 5 times more likely to develop psoriasis (OR = 5, 95% CI = 2.7 to 9.1). For the C/T genotype, it was 5 times more likely to develop psoriasis under over-dominant comparison (OR = 5, 95% CI = 2.59 to 9.4). Finally, allele T for this polymorphism was 3 times more likely to develop psoriasis under allelic comparison (OR = 3, 95% CI = 1.88 to 4.95) See Table 4.

### 3.5. Association of PSORS1 Locus Haplotypes with Psoriasis Severity

Gene–gene interaction analysis revealed that carriers for GGC genotype combinations had −0.73 times lower disease severity. On the contrary, carriers for GGT genotype combinations had −0.92 times more severe disease form (Table 5). These findings were based on SNPStats [19] web-based platform results, where descriptive statistics were used to estimate the relative frequency for each haplotype. Cumulative frequencies were also calculated to help in the selection of the threshold cut point to group rare haplotypes. The association analysis of haplotypes was either presented using logistic regression results with OR and 95% CI or linear regression results with differences in means and 95% CI. The most frequent haplotype was automatically selected as the reference category and rare haplotypes were pooled together in a group.

### 3.6. Relative Expression Analysis of Plasma PSORS1C1 in Psoriasis

**Figure 4:**
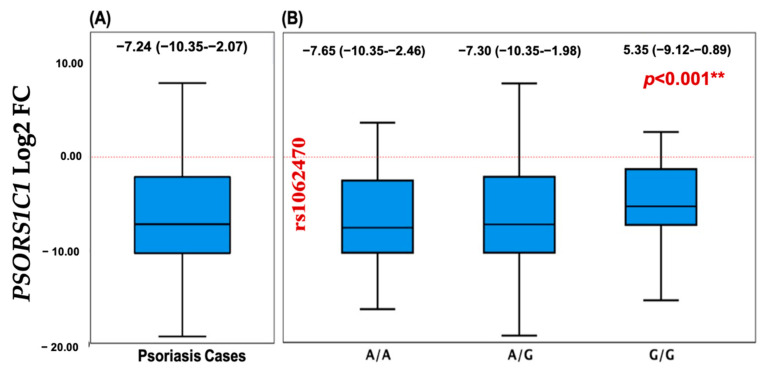
The relative expression profile of the *PSORS1C1* gene in psoriasis plasma samples. Data are shown as medians and quartiles. Box plot values were log-transformed, as data was non-parametric. The red dotted line represents the control level. Mann–Whitney U and Kruskal–Wallis tests were applied. (**A**) Overall psoriatic samples. (**B**) Stratified by rs1062470 genotype. ** Indicate highly significant value.

The relative gene expression of plasma *PSORS1C1* for psoriatic patients showed to be under-expressed compared to normal controls with log-transformed values for median and quartile levels equivalent to −7.24 (−10.35–−2.07) (Figure 4A). There were also significant differential expression levels among *PSORS1C1* SNP rs1062470 genotypes (*p* < 0.001) (Figure 4B).

### 3.7. Association of the Studied SNPs, PSORS1C1 Gene Expression, and Clinicopathological Features

**Figure 5:**
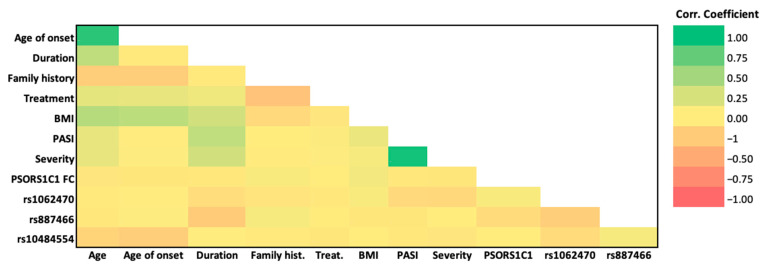
Heatmap presents the inter-relationship among the studied SNPs, *PSORS1C1* gene expression, and clinicopathological features. (BMI, Body Mass Index; PASI, Psoriasis Area and Severity Index).

A heatmap of the inter-relationship between the studied SNPs, *PSORS1C1* gene expression, and clinicopathological features is presented in Figure 5, and the correlation matrix in Table 6. Age was directly and significantly correlated with age of onset (r = 0.896; *p* < 0.001 ***), BMI (0.416; *p* < 0.001 ***), and duration (r = 0.367; *p* < 0.001***). BMI was directly and significantly correlated with age (0.416; *p* < 0.001 ***), age of onset (0.375; *p* < 0.001 ***), and duration (0.279; *p* < 0.005 **). PASI showed a highly significant, direct and very strong correlation with severity and vice versa (0.993; *p* < 0.001 ***).

## 4. Discussion

To date, there was only one comprehensive genome-wide association study (GWAS) done on the Egyptian population [22], identifying an association between MHC SNPs and psoriasis in a large Egyptian cohort, however, no data was reported from this study on the PSORS1 locus SNPs. The summary of the estimated genotype and allele frequency for each studied SNP based on worldwide previous publications was presented in Table 7.

In this study, we found significant associations for the three selected SNPs located within the PSORS1 locus with psoriasis risk and severity, both in single SNP and gene-gene interactions haplotype approaches. Table 5 describes the average genotype and allele frequency for each studied SNP based on the level of evidence demonstrated by a number of publications that included each SNP.

The rs10484554 genetic variant within *LOC105375015* was the most significantly associated with the disease, similar to that reported by Liu et al. [23], Képíró et al. [24], Kisiel et al. [25], Villarreal-Martínez et al. [26], and Strange et al. [27]. Our study odds ratio for the rs10484554 (T) minor allele (OR = 3, 95% CI = 1.88–4.95) was nearly the same as previously reported by Wiśniewski et al. [19] (OR = 2.68) and Villarreal-Martínez et al. [26] (OR=3). In comparing the minor allele frequency (MAF) for genetic variant (T) with other populations, the psoriatic patients in our study reported a MAF = 0.35, which is significantly higher than that reported worldwide (0.11); among Africans (0.08), Americans (0.08), East Asians (0.05), Europeans (0.14), and South Asians (0.2) [15]. In a comparison of different genotypes for this polymorphism with previous studies, the C/T genotype in our study was 5.63 times more likely to develop psoriasis under codominant comparison (OR = 1.84, 95% CI = 2.9–10.9); this is a much higher risk in comparison to Wiśniewski et al. [19] (OR = 3.38, 95% CI = 2.53–4.52). In contrast, the T/T genotype was 2.92 times more likely to develop psoriasis under codominant comparison (OR = 2.92, 95% CI = 0.97–8.8), and this is much lower than that reported by Wiśniewski et al. [19] (OR = 6.82, 95% CI = 4.11–11.30). In line with earlier publications [23,24,28,29,30,31], rs10484554 SNP in our study showed a significant correlation with early disease onset. This repetitive finding suggests that rs10484554 could play a role in early-onset psoriasis. However, Hébert et al. revealed that HLA-Cw*06 is associated with late-onset psoriasis using dense genotyping [32]. From a gene perspective, there are no published data about the possible role of *LOC105375015* in the pathogenesis of psoriasis. As rs10484554 belongs to a gene encoding lncRNA-*LOC105375015*—we assume that it might predispose to psoriasis via interacting with mRNA, DNA, protein and miRNA and consequently regulate gene expression at the epigenetic, transcriptional, post-transcriptional, translational, and post-translational levels in a variety of ways [33]. The function of this SNP is also unknown, but being in close proximity to the exon/intron junction, this may suggest a role in the splicing process. However, an explanation of this point requires further studies.

The genetic variant rs887466 from *PSORS1C3* was the only SNP associated with a protective effect in our study. We observed a protective effect only for the G/G genotype, which was 0.4 times more likely to protect against psoriasis under codominant comparison (OR = 0.4, 95% CI = 0.17 to 0.95) and recessive comparison (OR = 0.5, 95% CI = 0.23 to 1.05). In contrast, Wiśniewski et al. [19] reported that the A/A genotype was the protective one. Moreover, allele (G) for this polymorphism was 0.66 times more likely to protect against psoriasis under allelic comparison (OR = 0.66, 95% CI = 0.44 to 0.99), while Wiśniewski et al. [19] reported that the minor allele (A) was 0.77 times more protective against psoriasis, and De Bakker et al. [34] reported that the (G) allele was 5 times more likely to predispose to psoriasis and is considered a risk allele. Thus, the question is whether the (A) or (G) allele is the true marker of protection involving the *PSORS1C3* gene and is still to be investigated in future studies. In comparing the MAF for this genetic variant (A) with other populations, the psoriatic patients in our study reported a MAF = 0.62. This is higher than that reported worldwide (0.43); among Africans (0.37), Americans (0.51), East Asians (0.48), Europeans (0.41), and South Asians (0.41) [15]. The function of the relatively novel *PSORS1C3* gene is still under investigation. Specifically, nothing is known about the role of intronic rs887466. Previously, several SNPs in this gene have been tested in psoriasis in Swedish and Chinese populations [35,36], but rs887466 was just examined once in psoriasis. In our study, we found no significant association between rs887466 SNP and gender of patients or family history, contrasting Wiśniewski et al. [19]. In addition, when we stratified rs887466 SNP genotypes by both the age and gender of patients, we did not find any significant association between them, except with females aged from 41 to 50 years old. In consistence with Wiśniewski et al. [19], we found no statistically significant association between genotype frequencies of the rs887466 polymorphism and PASI score in both genders.

For the rs1062470 genetic variant, only the A/G genotype was 1.84 times more likely to develop psoriasis under over-dominant comparison (OR = 1.84, 95% CI = 1.09 to 3.13); other genotypes did not show a significant effect on disease risk. These results are contradicting those reported by Lesueur et al. [37] and Wiśniewski et al. [19] who stated that the AA genotype increased the risk of psoriasis over fivefold and was significantly associated with higher PASI score in males and explained it by the double effect of the (A) allele in the AA genotype, that may potentially elevate the expression of corneodesmosin in the skin and may result in increased severity of psoriasis. However, they were not able to explain why this effect was observed in males only. In comparing the MAF for this genetic variant (A) with other populations, the psoriatic patients in our study reported a MAF = 0.53, which is considered close to the reported range worldwide (0.45); among Africans (0.62), Americans (0.4), East Asians (0.51) and higher than that of the Europeans (0.35), and South Asians (0.31) [15].

We further assessed the association between the rs1062470 genetic variant and disease severity, and we observed a statistically significant association between rs1062470, and gender stratified by PASI. Interestingly, Wiśniewski et al. [19], Sakai et al. [38], and Hägg et al. [39] also observed that male patients, independently of rs1062470 genotype, had significantly higher PASI scores than female patients except for the earliest onset of disease. Our study is among few available studies describing a possible gender-dependent association of the rs1062470 genotype with psoriasis severity worldwide and the first to report this finding in Egypt; therefore, further larger-scale studies are needed, including also other polymorphisms in the *CDSN* gene, to confirm our findings. In addition, corneodesmosin expression levels in the psoriatic skin in both genders should be compared and its correlation with the rs1062470 genotype is mandatory to evaluate the gender-dependent effect of this SNP on disease severity.

In haplotype analysis, we observed that carriers for GGC genotype combinations had lower disease severity and carriers for GGT genotype combinations had a more severe disease form. Unfortunately, we were unable to determine whether these haplotypes correspond to other *HLA-C* alleles.

Although the PSORS1C1 gene is located within the PSORS1 locus and is considered one of the potential psoriasis susceptibility genes, the function of its gene product remains unclear in this disease. The relative gene expression level of the *PSORS1C1* gene in our cohort was significantly under-expressed, as shown in Figure 4A, opposing the expected assumption to be over-expressed. This can be justified by our cohort exclusion criteria, where we excluded psoriatic arthritis patients so as to decrease the study confounders. Our justification was based on Sun et al.’s findings which reported over-expression of the *PSORS1C1* gene in blood and synovial tissues [40], supporting the hypothesis that *PSORS1C1* plays a role in rheumatoid arthritis (RA) and is not in close association with the known HLA alleles.

In conclusion, our results demonstrated that rs10484554, rs887466 and rs1062470 genetic variants within the PSORS1 locus encompassing the multiple genes *LOC105375015, PSORS1C3* and *PSORS1C1/CDSN,* respectively, are significantly associated with psoriasis. This association is strongly dependent on genotype and less frequently the patient’s gender. Because of the complicated and extended LD pattern present in the MHC region, it is not clear whether the markers tested in this study confer the risk of psoriasis dependently or independently of other variants in this region. Our allelic discrimination analysis and association with disease or with the PASI score indicated the possibility that rs10484554 has a higher effect than rs887466 and rs1062470 on the risk and severity of psoriasis, and it is the major key player genetic variant in the PSORS1 locus. Finally, our results suggest that *PSORS1C1* gene under-expression might be in psoriatic patients free from arthritis. However, confirmation of this requires additional studies among Egyptians and other populations. Moreover, the functional consequence of the polymorphism needs to be investigated. Correlation analysis between the single peptide variant and expression levels in patients will add value to future studies outcomes.

## Figures and Tables

**Table 1:** Baseline characteristics among the study population.

	Cases (*n* = 100)		Control (*n* = 100)		*p*
	No.	%	No.	%	
Age (years)					
● Min.–Max.	18.0–60.0		20.0–62.0		0.134
● Mean ± SD.	41.74 ± 14.08		39.17 ± 11.65		
● Median (IQR)	42.0 (31.50–56.0)		37.0 (30.0–49.0)		
Gender					
● Male	47	47.0	55	55.0	0.258
● Female	53	53.0	45	45.0	
Special habits					
● Non-smoker	77	77.0	72	72.0	0.417
● Smoker	23	23.0	28	28.0	
BMI (kg/m^2^)					
● Min.–Max.	19.0–36.21		19.55–35.63		0.112
● Mean ± SD.	26.80 ± 3.99		27.70 ± 3.93		
● Median (IQR)	25.91 (24.13–29.45)		27.43 (25.0–30.8)		

Data are shown as number (percentage) or mean ± SD. *p*-value < 0.05 was considered as statistically significant.

**Table 2:** Disease characteristics among psoriatic study population (*n* = 100).

Disease Characteristic	No.	%
**Age of onset**		
● vEOP	21	21.0
● mEOP	42	42.0
● LOP	37	37.0
● Min.–Max.	3.0–59.0	
● Mean ± SD.	35.07 ± 13.43	
● Median (IQR)	36.0 (24.13–29.45)	
**Severity**		
● Mild	45	45.0
● Moderate	31	31.0
● Severe	24	24.0
**Duration (years)**		
● Min.–Max.	0.50–30.0	
● Mean ± SD.	6.75 ± 6.22	
● Median (IQR)	5.0 (25.0–30.80)	
**Family history**		
● No	84	84.0
● Yes	16	16.0
**Treatment**		
● No Treatment	41	41.0
● On Treatment	59	59.0

Data are shown as number (percentage) or mean ± SD; vEOP: very early-onset psoriasis; mEOP: middle early-onset psoriasis; LOP: late-onset psoriasis.

**Table 3:** Relation between age of onset with gender and PASI in patient group (*n* = 100).

Age of Onset	vEOP (*n* = 21)		mEOP (*n* = 42)		LOP (*n* = 37)		*p*
	No.	%	No.	%	No.	%	
**Gender**							
- **Male**	3	14.3	22	52.4	22	59.5	0.003 *
- **Female**	18	85.7	20	47.6	15	40.5	
**PASI**							
- **Min.–Max.**	3.50–38.30		2.0–40.50		1.50–35.60		0.564
- **Mean ± SD.**	12.28 ± 7.84		14.72 ± 9.56		13.29 ± 9.31		
- **Median**	11.50		12.25		12.50		

Data are shown as number (percentage); *p*: *p*-value for comparing between the studied groups; *: statistically significant at *p* ≤ 0.05 psoriasis; vEOP: very early-onset psoriasis; mEOP: middle early-onset psoriasis; LOP: late-onset psoriasis.

**Table 4:** Genetic Association Models for *PSORS1C3* Gene Polymorphism with Disease Risk.

SNP	Model	Genotype	Patients	Controls	OR (95% CI)	*p*-Value
**rs887466**	Codominant	A/A	36	26	Reference	
		A/G	51	51	0.72 (0.38–1.36)	0.3
		G/G	13	23	0.4 (0.17–0.95)	0.038 *
	Dominant	A/A	36	26	Reference	
		A/G-G/G	64	74	0.62 (0.34–1.14)	0.13
	Recessive	A/A-A/G	87	77	Reference	
		G/G	13	23	0.5 (0.23–1.05)	0.068
	Over-dominant	A/A-G/G	49	49	Reference	
		A/G	51	51	1.0 (0.57–1.74)	1.0
	Allelic Model	A	123	103	Reference	
		G	77	97	0.66 (0.44–0.99)	0.04 *
**rs1062470**	Codominant	A/A	25	25	Reference	
		A/G	56	53	1.05 (0.54–2.06)	0.87
		G/G	19	22	0.86 (0.37–1.97)	0.72
	Dominant	A/A	25	25	Reference	
		A/G-G/G	75	75	1.04 (0.66–1.62)	0.87
	Recessive	A/A-A/G	81	78	Reference	
		G/G	19	22	0.83 (0.41–1.65)	0.6
	Over-dominant	A/A-G/G	44	77	Reference	
		A/G	56	53	1.84 (1.09–3.13)	0.02 *
	Allelic Model	A	106	103	Reference	
		G	94	97	0.94 (0.63–1.39)	0.76
**rs10484554**	Codominant	C/C	39	76	Reference	
		C/T	52	18	5.63 (2.9–10.9)	<0.001 *
		T/T	9	6	2.92 (0.97–8.8)	0.05 *
	Dominant	C/C	39	76	Reference	
		C/T-T/T	61	24	5 (2.7–9.1)	<0.001 *
	Recessive	C/C-C/T	91	94	Reference	
		T/T	9	6	1.54 (0.53–4.52)	0.42
	Over-dominant	C/C-T/T	48	82	Reference	
		C/T	52	18	5 (2.59–9.4)	<0.001 *
	Allelic Model	C	130	170	Reference	
		T	70	30	3 (1.88–4.95)	<0.001 *

A: Adenine; C: Cytosine; CI: Confidence Interval; G: Guanine; OR: Odds Ratio; T: Thymine; *p*: *p*-value for comparing between the studied groups; *: Statistically significant at *p* ≤ 0.05.

**Table 5:** Haplotype association with Disease Severity.

	rs887466	rs1062470	rs10484554	Frequency	OR (95% CI)	*p*-Value
**1**	G	A	C	0.199	Reference	-
**2**	A	G	C	0.1954	NA (NA–NA)	NA
**3**	A	A	C	0.1672	0.4 (−0.06–0.85)	0.089
**4**	A	G	T	0.1508	0.3 (−0.12–0.72)	0.16
**5**	A	A	T	0.1015	−0.36 (−0.77–0.04)	0.082
**6**	G	G	C	0.0884	−0.73 (−1.21–−0.24)	0.0038 *
**7**	G	A	T	0.0623	−0.32 (−0.8–0.17)	0.2
**8**	G	G	T	0.0354	0.92 (0.18–1.66)	0.015 *

A: Adenine; C: Cytosine; CI: Confidence Interval; G: Guanine; NA: Not Applicable; OR: Odds Ratio; T: Thymine; *p*: *p*-value for comparing between the studied groups; *: Statistically significant at *p* ≤ 0.05.

**Table 6:** Correlation matrix showing the inter-relationships among the studied SNPs, *PSORS1C1* gene expression, and clinicopathological features.

		Age	Age of Onset	Duration	Family History	Treatment	BMI	PASI	Severity	*PSORS1C1* FC	rs1062470	rs887466	rs10484554
**Age**	R	1	0.896 **	0.367 **	−0.237 *	0.175	0.416 **	0.163	0.164	−0.056	−0.021	−0.039	−0.184
	P	.	<0.001	<0.001	0.018	0.083	<0.001	0.106	0.103	0.583	0.832	0.698	0.067
**Age of onset**	R	0.896 **	1	−0.023	−0.225 *	0.158	0.375 **	0.022	0.054	−0.05	0.022	0.046	−0.227 *
	P	<0.001	.	0.824	0.024	0.118	<0.001	0.831	0.596	0.624	0.83	0.653	0.023
**Duration**	R	0.367 **	−0.023	1	−0.017	0.125	0.279 **	0.354 **	0.275 **	−0.034	−0.111	−0.251 *	0.033
	P	<0.001	0.824	.	0.866	0.218	0.005	<0.001	0.006	0.736	0.27	0.012	0.745
**Family history**	R	−0.237 *	−0.225 *	−0.017	1	−0.310 **	−0.15	0.005	−0.005	0.075	−0.071	0.084	−0.018
	P	0.018	0.024	0.866	.	0.002	0.147	0.963	0.96	0.46	0.483	0.407	0.855
**Treatment**	R	0.175	0.158	0.125	−0.310 **	1	−0.06	0.062	0.056	0.027	−0.038	−0.013	−0.046
	P	0.083	0.118	0.218	0.002	.	0.578	0.542	0.581	0.788	0.706	0.9	0.649
**BMI**	R	0.416 **	0.375 **	0.279 **	−0.146	−0.057	1	0.147	0.082	0.118	0.076	−0.051	−0.001
	P	<0.001	<0.001	0.005	0.147	0.578	.	0.145	0.417	0.242	0.454	0.612	0.994
**PASI**	R	0.163	0.022	0.354 **	0.005	0.062	0.147	1	0.930 **	−0.037	−0.134	−0.049	−0.038
	P	0.106	0.831	<0.001	0.963	0.542	0.145	.	<0.001	0.716	0.183	0.63	0.705
**Severity**	R	0.164	0.054	0.275 **	−0.005	0.056	0.082	0.930 **	1	−0.051	−0.156	0.015	−0.062
	P	0.103	0.596	0.006	0.96	0.581	0.417	<0.001	.	0.611	0.12	0.881	0.539
***PSORS1C1* FC**	R	−0.056	−0.05	−0.034	0.075	0.027	0.118	−0.037	−0.051	1	0.074	−0.126	0.008
	P	0.583	0.624	0.736	0.46	0.788	0.242	0.716	0.611	.	0.465	0.212	0.938
**rs1062470**	R	−0.021	0.022	−0.111	−0.071	−0.038	0.076	−0.134	−0.156	0.074	1	−0.231 *	−0.127
	P	0.832	0.83	0.27	0.483	0.706	0.454	0.183	0.12	0.465	.	0.021	0.209
**rs887466**	R	−0.039	0.046	−0.251 *	0.084	−0.013	−0.05	−0.049	0.015	−0.126	−0.231 *	1	0.081
	P	0.698	0.653	0.012	0.407	0.9	0.612	0.63	0.881	0.212	0.021	.	0.425
**rs10484554**	R	−0.184	−0.227 *	0.033	−0.018	−0.046	−0	−0.038	−0.062	0.008	−0.127	0.081	1
	P	0.067	0.023	0.745	0.855	0.649	0.994	0.705	0.539	0.938	0.209	0.425	.

Correlation coefficient (R) represents the value for Spearman’s correlation analysis and its *p*-values (P). Shaded boxes enclose values which are statistically significant at either *p* < 0.05 (*) or *p* < 0.01 (**). Abbreviations: BMI, Body Mass Index; FC, Fold Change; PASI, Psoriasis Area and Severity Index.

**Table 7:** Summary of the estimated genotype and allele frequency for each studied SNP based on worldwide previous publications (Data adopted from https://opensnp.org/ (accessed on 1 March 2022) [18]).

SNP	Genotype Frequency	Allele Frequency	Level of Evidence
rs1062470	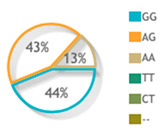	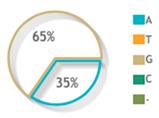	4 publications
rs887466	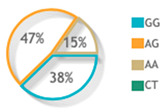	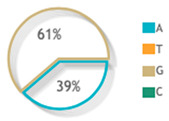	7 publications
rs10484554	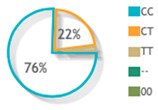	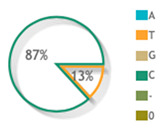	39 publications

## Data Availability

The data that supports the findings of this study are available from the corresponding author upon reasonable request.
